# Selective attention affects conceptual object priming and recognition: a study with young and older adults

**DOI:** 10.3389/fpsyg.2014.01567

**Published:** 2015-01-12

**Authors:** Soledad Ballesteros, Julia Mayas

**Affiliations:** Studies on Aging and Neurodegenerative Diseases Research Group, Department of Basic Psychology II, Universidad Nacional de Educación a DistanciaMadrid, Spain

**Keywords:** aging, implicit memory, explicit memory, recognition, conceptual repetition priming, selective attention

## Abstract

In the present study, we investigated the effects of selective attention at encoding on conceptual object priming (Experiment 1) and old–new recognition memory (Experiment 2) tasks in young and older adults. The procedures of both experiments included encoding and memory test phases separated by a short delay. At encoding, the picture outlines of two familiar objects, one in blue and the other in green, were presented to the left and to the right of fixation. In Experiment 1, participants were instructed to attend to the picture outline of a certain color and to classify the object as natural or artificial. After a short delay, participants performed a natural/artificial speeded conceptual classification task with repeated attended, repeated unattended, and new pictures. In Experiment 2, participants at encoding memorized the attended pictures and classify them as natural or artificial. After the encoding phase, they performed an old–new recognition memory task. Consistent with previous findings with perceptual priming tasks, we found that conceptual object priming, like explicit memory, required attention at encoding. Significant priming was obtained in both age groups, but only for those pictures that were attended at encoding. Although older adults were slower than young adults, both groups showed facilitation for attended pictures. In line with previous studies, young adults had better recognition memory than older adults.

## INTRODUCTION

The aim of the present study was to investigate the effects of aging and selective attention at encoding on two types of long-term memory: repetition priming as a measure of implicit memory, assessed by showing priming effects in a speeded conceptual classification task (Experiment 1), and explicit (episodic) memory, assessed by an old–new recognition task (Experiment 2). The procedures of both experiments included an encoding phase and a memory test phase, separated by 3 min performing a distraction task. Both experiments were administered during functional magnetic resonance imaging (fMRI) scanning (fMRI results will be reported separately).

Implicit memory, a type of indirect, unintentional manifestation of prior experience, is often demonstrated by showing repetition priming. Repetition priming is a change in speed or accuracy when processing previously encountered stimuli compared to stimuli not previously encountered, even without awareness that the stimulus has been experienced before (Tulving and Schacter, 1990). Priming can also result in changes in response bias that does not change overall accuracy. Implicit memory has been contrasted with explicit memory, the conscious or intentional retrieval of past experience (Schacter, 1987). It is well documented in the literature that explicit memory declines with age. Memory declines occur later and are less pronounced in longitudinal (e.g., Nilsson, 2003; Rönnlund et al., 2005, 2008) than cross-sectional (Park et al., 2001) studies. Episodic (explicit) memory involves the retrieval of a stimulus with information about the context in which it was encountered (Tulving, 2002). Numerous studies have shown that implicit memory is spared in older adults (e.g., Mitchell, 1989; Mitchell and Bruss, 2003; Ballesteros and Reales, 2004; Wiggs et al., 2006; Ballesteros et al., 2007, 2009; for reviews see Fleischman and Gabrieli, 1998; Fleischman, 2007), although some studies have reported age differences in priming (e.g., La Voie and Light, 1994; Keane et al., 2004; Wiggs et al., 2006; Gordon et al., 2013; Word et al., 2013).

It is well established in the literature that perceptual (data-driven) and conceptual (conceptually driven) priming can be dissociated (e.g., Cabeza and Ohta, 1993; Gabrieli et al., 1994; Lyttle et al., 2010). Indirect memory tests based on conceptual processes require the extraction and retention of the meaning of the stimuli, whereas indirect tests based on perceptual processes require the analysis and processing of their surface features.

Selective attention refers to the process of centering resources on certain stimuli or on specific aspects of the input. Thus, attended information is selected and processed further while unattended input is filtered out. Divided attention refers to the efficient allocation of resources between different stimuli by shifting the attentional focus. It has been well established that attention at encoding is required for episodic memory (e.g., Rock and Gutman, 1981; Craik et al., 1996; Mulligan, 1998). A number of early studies using the divided attention paradigm showed negative effects on explicit memory tasks and very little or no effect on implicit memory tests (e.g., Parkin and Russo, 1990; Szymanski and MacLeod, 1996). However, a recent review and meta-analysis including 38 effect sizes extracted from 21 empirical studies indicated that divided attention produced a small, but significant, negative effect on implicit memory (Spataro et al., 2011). Spataro et al. (2011) examined the potential contamination of implicit memory by deliberate recollection and concluded that the results do not support the hypothesis that divided attention effects on implicit memory are due to explicit contamination.

Further studies reported attentional effects for words in both explicit and implicit memory tests (e.g., MacDonald and McLeod, 1998; Crabb and Dark, 1999; Stone et al., 2000). In a recent study, Prull (2013) showed that verb generation (a conceptual implicit memory task) is sensitive to divided attention at encoding, because using a demanding distracting task reduced priming significantly, while the manipulation of selective attention eliminated priming. According to the literature, selective attention tends to produce stronger effects on memory than divided attention. Thus, the attentional manipulation used in the present study should reduce both implicit and explicit memory test performance.

Studies in our laboratory that used pictorial stimuli have investigated the role of selective attention at encoding in perceptual priming tasks in children, young adults, and older adults (Ballesteros et al., 2006, 2007, 2008). These studies assessed implicit memory with *picture fragment completion* and *speeded object naming* tasks. These tasks are considered to be perceptual priming tasks, although *object naming* appears to be more complex, with both perceptual and conceptual contributions to the observed priming effect (Bruss and Mitchell, 2009).

Perceptual repetition priming of pictures is less sensitive than explicit memory to limited attentional resources, but both types of memory require attention at encoding. In a previous study, we (Ballesteros et al., 2008) used overlapping picture outlines at encoding to investigate the effect of selective attention on perceptual repetition priming. Young adults, cognitively normal older adults and patients with Alzheimer’s disease (AD) were shown picture outlines (visual modality, Experiment 1) and objects presented to touch without vision, one to the attended hand and another to the unattended hand (haptic modality, Experiment 2). Similar results were found in the three groups. Young and cognitively normal older adults showed similar priming in the two modalities (vision and touch) but only for those stimuli that were attended at encoding. In contrast, we did not find priming for stimuli that were unattended at encoding. The results suggest that in both perceptual modalities, repetition priming requires attention at encoding in both age groups. Older adults, although slower, showed the same perceptual facilitation as young adults, but only for attended stimuli. However, AD patients did not show priming for either attended or unattended stimuli, suggesting an early deficit of selective attention due perhaps to visual clutter arising from their reduced ability to filter out irrelevant information (Hasher and Zacks, 1988) produced by the overlapping of the picture outlines.

To avoid visual clutter, in further studies we used a modified selective attention procedure at encoding in which, instead of presenting two overlapping picture outlines, the two outlines (one attended and the other unattended) were presented about 6 cm apart, one on the left and the other on the right of fixation (Ballesteros et al., 2013b,c). The results did not show perceptual facilitation for unattended stimuli in either sedentary or physically active older adults (Ballesteros et al., 2013b). Consistent with previous results, young adults and cognitively normal older adults showed priming for attended pictures but not for unattended ones, while older adults with mild cognitive impairment (MCI) showed a lack of visual priming even for attended stimuli (Ballesteros et al., 2013c). We postulated that the lack of priming when attention was compromised at study might be a marker of pathological aging.

We investigated two main questions in the present study. First, we investigated whether the deployment of attention to an object at encoding is required for conceptual repetition priming. Second, we examined whether cognitively normal older adults would perform worse than younger adults not only in the explicit memory task but also in the conceptual priming task for attended objects. Older adults have reduced ability to filter out irrelevant information (e.g., Hasher and Zacks, 1988; Weeks and Hasher, 2014). Consequently, it could be expected that older adults would show some priming for unattended objects. In sum, we expected significant priming in both young and older adults for attended pictures, but possibly reduced in the older group. We did not expect priming for the pictures that were unattended at encoding in the young adult group, but some priming could be observed for unattended stimuli in the older group.

In the present study, we used a selective attention paradigm with pictures presented to the left and right of fixation, which were either attended or unattended. The encoding phases of the conceptual object priming and explicit recognition tests followed a study-test paradigm and used similar types of stimuli. Participants in the conceptual priming task had to classify the object outlined in a predetermined color as quickly as possible. At test, a single black outline picture was presented at each trial with specific instructions for each type of memory test. The same young and older adults participated in the two experiments. The conceptual priming experiment was always performed first to avoid contamination of implicit memory by explicit memory. Experiment 1 evaluated the effect of selective attention at encoding on an incidental conceptual priming task. After an encoding phase, participants performed a speeded natural/artificial classification task with picture outlines of familiar objects. Experiment 2 explored the effect of aging and selective attention at encoding on an explicit old–new recognition task.

## MATERIALS AND METHODS

The current study was approved and conducted in compliance with the guidelines set out by the Ethical Review Board of the* Universidad Nacional de Educación a Distancia* (*UNED*). All participants gave their written informed consent before the study started and were informed of their right to terminate participation in the study at any time. The study was in accordance with the ethical standards laid down in the 1964 Declaration of Helsinki. Both experiments began with a study phase, followed after a short resting interval of ∼3 min by the test phase. Participants performed both experiments inside the MRI scanner.

### PARTICIPANTS

There were 24 young adults (mean age = 30 years; SD = 4.36; range = 21–39 years; 8 males and 16 females) and 20 cognitively normal older adults (mean age = 69; SD = 4.18; range = 63–76 years; 9 males and 11 females) who participated voluntarily in this study. All participants were right-handed. All the older adults showed normal mental functioning [mini-mental state examination (MMSE)- score > 27]. All had normal or corrected-to-normal vision and normal color vision. Younger and older adults did not differ significantly with regard to the number of years of education, scores on the Vocabulary subtests of the Wechsler-III Battery (Wechsler, 1999; Spanish version by Wechsler and Pereña, 2004) or the Yesavage Geriatric Depression Scale (Yesavage et al., 1983; Spanish adaptation by Martínez et al., 2002). However, they differed in the MMSE (Folstein et al., 1975). The results are displayed in **Table 1** and are within the range of performance associated with normal age-related cognitive differences. All participants were given an optometric examination prior to the scanning session. Visual acuity was corrected to 20/20 on the Snellen Scale. Participants used a pair of lenses mounted on the scanner goggles when needed, based on their optometric prescription. All participants gave informed consent for participation in the experiments.

**Table 1:** Mean scores of screening tests for the two groups and mean years of formal education (SD in parentheses).

Group	Age (year)	Education (year)	MMSE*	Yesavage	Vocabulary
Young adults (24)	30 (4.36)	14.6 (2.3)	29.7 (0.53)	0.87 (0.78)	59.12 (5.27)
Older adults (20)	69 (4.18)	13.8 (4.1)	28.6 (1.11)	1.95 (2.43)	62.72 (8.03)

*p < 0.05, T for independent samples. MMSE, mini mental state examination (/30); Yesavage, Geriatric Depression Scale (GDS).

### STIMULI AND EQUIPMENT

The stimuli used in the present study consisted of 240 picture outlines, 120 taken from Snodgrass and Vanderwart’s (1980) set and another 120 from Bonin et al. (2003). The total set of 240 stimuli was divided randomly into two sets of 120 stimuli each. One of the sets was used in Experiment 1 and the other in Experiment 2. Adobe Photoshop CC (Adobe Systems Software, Ireland Ltd.) was used to adjust the figure-ground of the two data sets to the same size (10 cm × 10 cm, approximately). We used Super-Lab 4.0 programming software (Cedrus Corporation, San Pedro, CA, USA) to design the experiments, display stimuli, control timing, and log participants’ responses through fMRI response pads. Stimuli were presented through optic-fiber-based glasses (MRVision 2000 ultra, Resonance Technology, Inc., Northridge, CA, USA) connected to the stimulation computer. Responses were registered with Lumina LP400 response pads for fMRI.

The stimuli were prepared in three different colors (blue, green, and black). Half of the stimuli depicted artificial objects (e.g., ball, cigarette, microphone, door) and the other half depicted natural objects (e.g., baby, orange, tree, lion). The pictures subtended a mean visual angle of ∼4.3 × 4.9°. At encoding, the two pictures presented were shown inside a box of 22.6 cm × 9.5 cm subtending a visual angle of 8.5 × 16.5°. This box was used to focus attention. Pictures in each pair were 1 cm apart with fixed eccentricity. All the stimuli were presented in black on a white background.

### PROCEDURE

Before the experimental part of the study, participants completed a semi-structured clinical interview and performed a series of screening tests in a small interview room. This part of the study lasted ∼25 min. In each task (implicit and explicit), 120 stimuli were used. Half of the pictures in each list depicted natural objects and the other half artificial objects. One list was used in the implicit memory task and the other list in the explicit memory task. The stimuli and procedure in the two tasks were similar, the only difference being the instructions provided by the experimenter at encoding and test.

**FIGURE 1:**
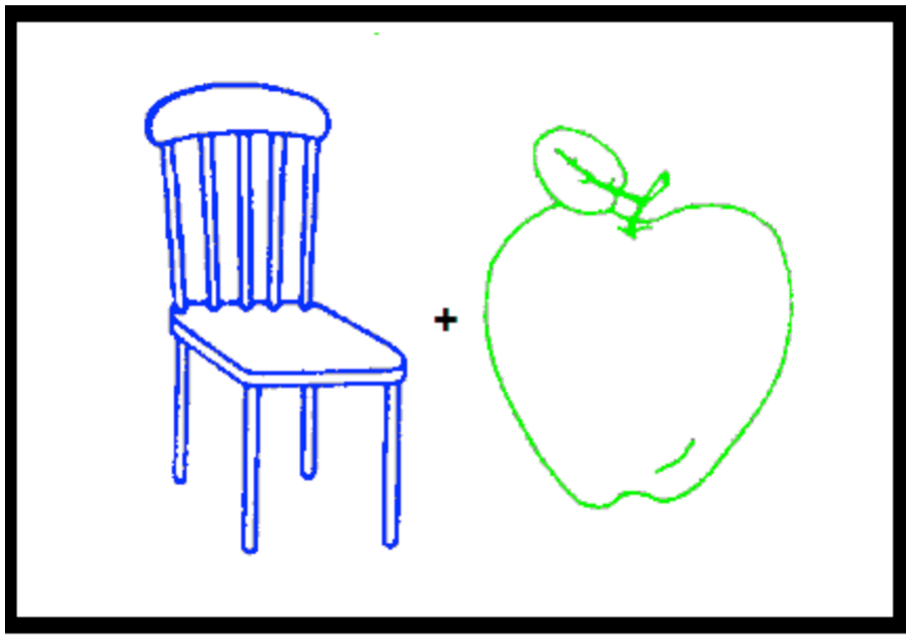
**An example of the two picture outlines, one green and the other blue, presented during the encoding phase of Experiments 1 and 2**.

Both experiments started with an encoding phase followed after a short delay by a test phase. At encoding (study phase), participants were presented with 36 trials. On each trial, two picture outlines were displayed 1 cm apart, one on the left and the other on the right of fixation to make sure that both stimuli entered the field of attention. One outline was green and the other was blue. Half of the participants attended the picture with the green outline and the other half to the picture with the blue outline. In half of the trials, the blue outline appeared on the left and in the other half on the right of fixation (see **Figure 1**). At test, the 72 stimuli presented at study plus 48 new picture outlines were presented in a random order that differed for each participant.

**FIGURE 2:**
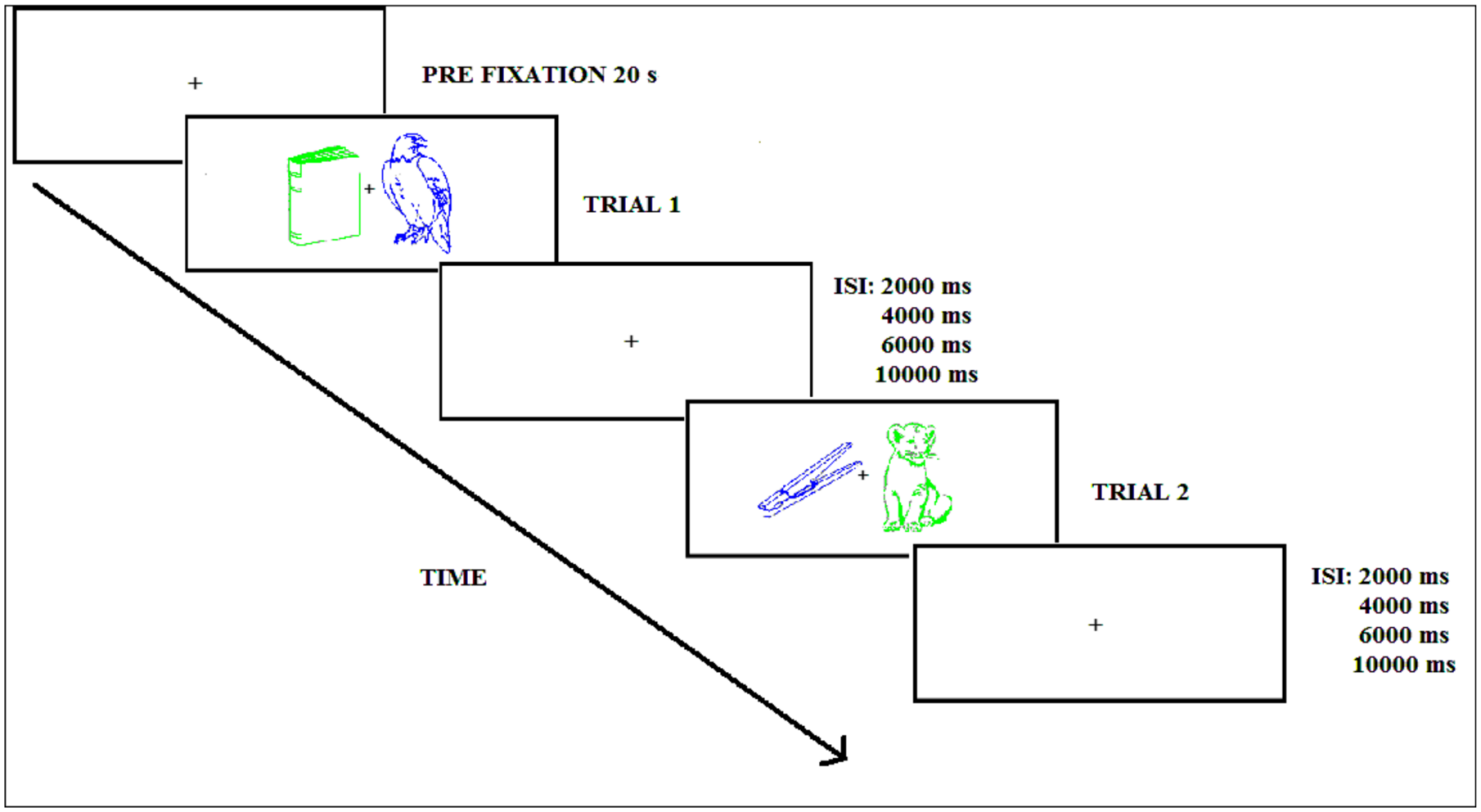
**Experimental procedure during the encoding phase (Experiments 1 and 2); ISI refers to the inter-stimulus interval**.

The trial sequence corresponding to the encoding phase in both Experiments is shown in **Figure 2** and started with a central white fixation cross appearing for 20 s. Next, the green and blue picture outlines were displayed to the left and right of fixation for 1000 ms, followed by a variable inter-stimulus interval (ISI: 2000, 4000, 6000, or 10000 ms) with an average of 6500 ms for attended and unattended stimuli. When this interval elapsed, the following pair of stimuli appeared to the left and right of fixation. Participants were instructed to pay attention to the picture of a given color (the attended color was counterbalanced across participants) and to disregard the other picture. The task consisted of pressing as fast as possible with the right or left hand a button on the response pad classifying the attended picture as “natural” or “artificial.” The response hand was counterbalanced across participants. In addition, in Experiment 2, participants had to classify the stimulus as natural/artificial and try to remember the picture. The participant was instructed to keep the gaze focused on the fixation cross. During the practice trials participants received visual feedback indicating whether the response was correct or incorrect, but no feedback was given during the experimental phases.

**FIGURE 3:**
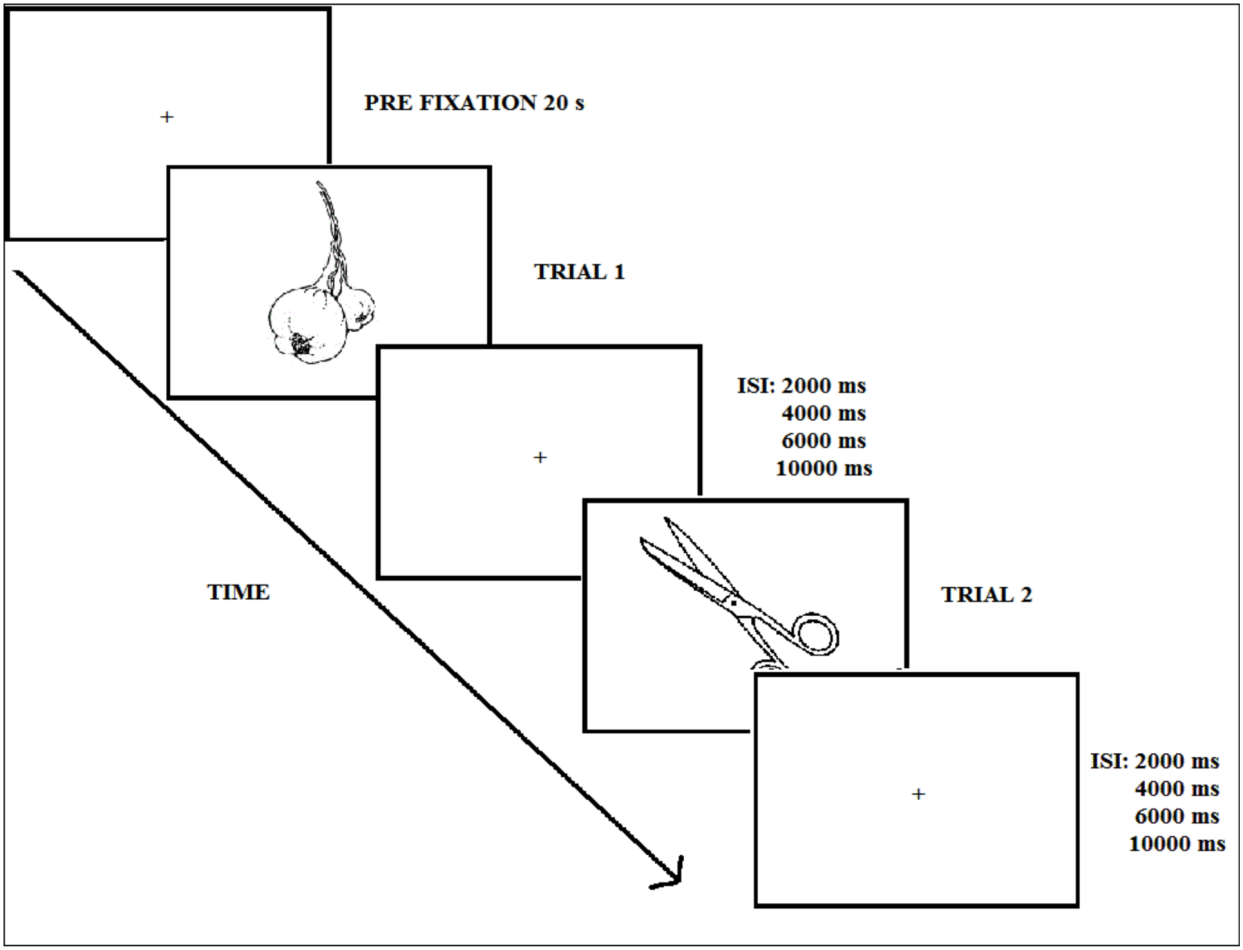
**Experimental procedure during the test phase (Experiments 1 and 2); ISI refers to the inter-stimulus interval**.

After a 3-min fluency task consisting of naming words starting by a certain letter (e.g., f, l, m), participants performed the implicit natural-artificial classification task incidentally (Experiment 1), or the old–new explicit recognition memory task (Experiment 2). The trial sequence corresponding to the test phase in the two experiments is displayed in **Figure 3**. At test, the 120 pictures were presented one by one in black.

## EXPERIMENT 1: IMPLICIT MEMORY

After the interview and screening tests, each participant was trained on the task for ∼15 min in a quiet room before the implicit memory test started. Participants completed 30 practice trials. None of the practice trials were presented at test. The implicit classification task lasted about 35 min and consisted of a study phase and a test phase as described above.

### EXPERIMENTAL DESIGN

The experimental design consisted of a 2 (group: young adults and older adults) × 3 (study condition: attended, unattended, and non-studied pictures) mixed factorial design. Group was the between-subjects factor and study condition was the within-subjects factor.

### ENCODING PHASE

As described above, participants performed the conceptual classification task on each of the 36 trials and were asked to respond as fast and accurately as possible.

### TEST PHASE

After the study phase, and when the 3 min (distraction task) had elapsed, participants performed the speeded natural/artificial classification task by pressing one of two keys. In this phase, participants were presented with the 72 studied pictures that had been displayed during the encoding phase (36 attended and 36 unattended), intermixed with 48 new pictures. The order of presentation of the 120 stimuli (36 attended, 36 unattended and 48 non-studied) was randomized for each participant.

The trial sequence started with a central white fixation cross appearing for 20 s, followed by the picture outline presented in the center of the screen. Participants categorized the picture as fast as possible. Latency was measured from the time the picture outline appeared on the screen until the participant’s response (see **Figure 2**). Performance was assessed by the response time at which the stimuli were correctly classified.

When the experiment ended, participants were asked if they were aware of the repetition of the stimuli. The results confirmed that none were aware that stimuli from the first phase of the experiment had been presented again.

### RESULTS AND DISCUSSION

To investigate whether the young and older adults were similarly accurate in the classification task at encoding, we calculated the mean number of errors of the older adults and the young adults. Four young adults were excluded from the analyses due to technical problems. Three older participants were also excluded due to their low accuracy (below 75% correct). So, 20 young adults and 17 cognitively normal older adults entered into the analyses. The mean number of errors of young adults and older adults were 1 and 2, respectively. The univariate ANOVA performed on accuracy using the number of errors as the dependent variable showed that the main effect of group was marginally significant [*F*(1,35) = 3.92, *p* = 0.05, MSE = 2.34, partial η^2^ = 0.10, 1-β error prob = 0,38].

**FIGURE 4:**
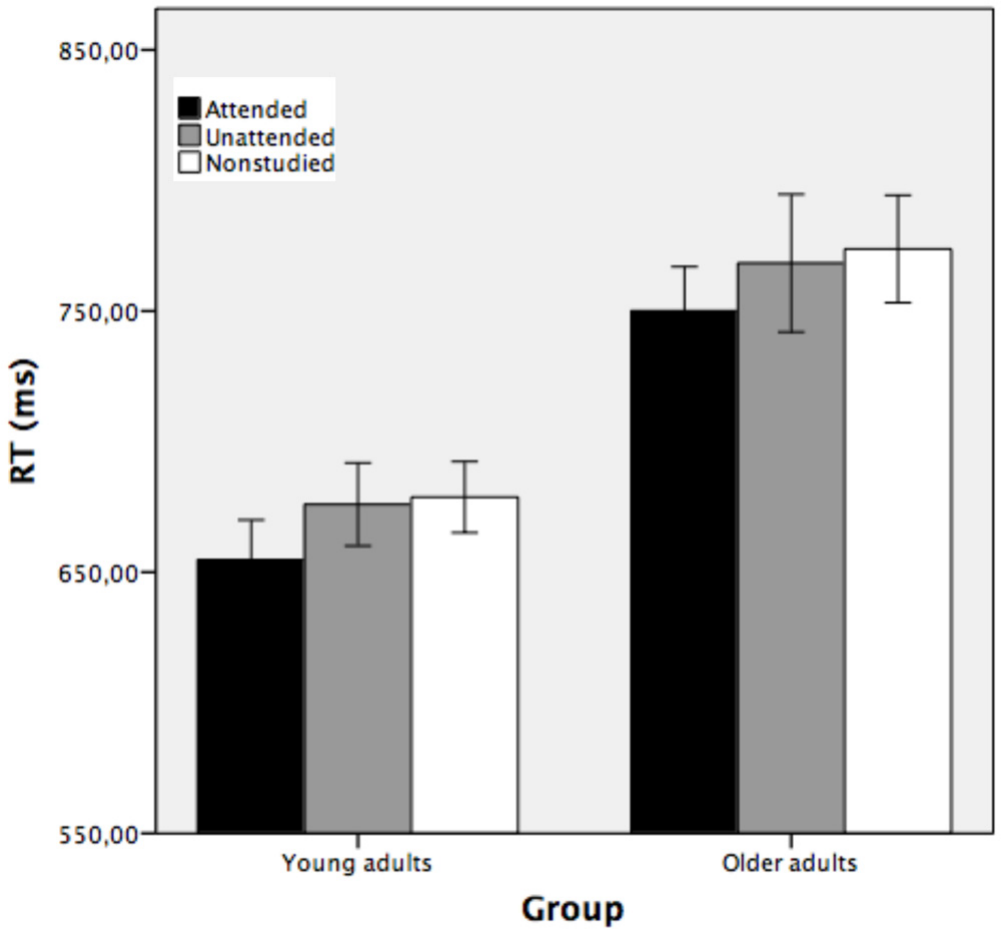
**Mean reaction time (in ms) obtained in the conceptual implicit memory task as a function of group (young and older adults), corresponding to attended, unattended and non-studied picture outlines (Experimrnt 1).** Bars indicate ±1 SD from the mean.

To assess conceptual repetition priming, separate ANOVAs were performed using the percentage of errors and RTs as dependent measures. Trials with reaction times (RTs) faster than 200 ms or slower than 2000 ms (3.5 and 4.6% for younger and older adults, respectively) were excluded from the analyses. **Figure 4** shows the average response time taken to classify the attended, unattended, and non-studied stimuli as a function of group and study condition.

The mixed factorial ANOVA performed on the percentage of errors with group as the between-subjects factor and study condition as the repeated measure showed that the main effect of study was marginally significant [*F*(2,70) = 2.78, MSE = 4.47, *p* = 0.06, partial η^2^ = 0.07, 1-β error prob = 0.61]. The mean percentage of errors was 2.34, 3.51, and 2.89 for attended, unattended and non-studied stimuli, respectively. The main effect of group was also significant [*F*(1,35) = 8.22, MSE = 10.35, *p* < 0.001, partial η^2^ = 0.19, 1-β error prob = 0.99]. The mean percentage of errors for young participants was 2.03%, while for older adults it was 3.79%. However, the two-way group × study condition interaction was not significant [*F*(2,70) = 2.6, MSE = 4.47, *p* > 0.05, partial η^2^ = 0.08, 1-β error prob = 0.91], suggesting that there was no age difference in the relative number of errors across the three conditions (attended, unattended and non-studied stimuli).

A mixed factorial ANOVA conducted on RTs with group as between-subjects factor and study condition as the repeated measure showed that the main effect of study was reliable [*F*(2,70) = 10.08, MSE = 594.60, *p* < 0.001, partial η^2^ = 0.22, 1-β error prob = 0.96]. Pairwise comparisons showed that attended stimuli were classified faster (*M* = 702 ms) than unattended (*M* = 722 ms, *p* < 0.05) and non-studied (*M* = 726 ms, *p* < 0.01) stimuli, while the two latter conditions did not differ significantly (*p* > 0.05). The main effect of group was statistically significant [*F*(1,35) = 14.37, MSE = 17020.59, *p* < 0.001, partial η^2^ = 0.29, 1-β error prob = 1], indicating that young participants categorized the stimuli faster (*M* = 670 ms) than the older adults (*M* = 764 ms). The two-way group × study condition interaction was not significant [*F*(2,70) = 0.04, MSE = 594.60, *p >* 0.05, partial η^2^ = 0.001, 1-β error prob = 0.06], suggesting that the two groups of participants showed similar conceptual indirect facilitation when the stimuli were attended at encoding, but there was no conceptual priming for objects that were unattended at encoding.

Conceptual repetition priming was shown by the difference between the time taken to classify correctly repeated outlines presented during the study phase (attended and unattended) and the time to classify the new (non-studied) stimuli. Young and older adults classified the attended stimuli faster than unattended and non-studied stimuli. Both groups of participants were faster under the attended condition (means 655 and 750 ms for young and older adults, respectively) than under the unattended condition (means 676 and 768 ms for young and older participants, respectively) and non-studied condition (means 679 and 774 ms for young and older participants, respectively). See **Figure 4**.

Given that there was an age difference in RT to new items, we report the difference measure as well as a proportional measure that takes into account the baseline difference between young and older adults. The proportional RT priming scores were computed using the corrected formula: [(Non-studied RT-Studied RT)/Non-studied RT) ∗ 100] for attended and unattended items. A *t-*test for independent samples using the transformed data showed that the priming scores of young and older adults for attended and unattended stimuli did not differ statistically [*t*(1,35) = 0.67, *p* > 0.05; *t*(1,35) = -0.31, *p* > 0.05].

The results of the young and older adults in this conceptual repetition priming task are in agreement with previous results with young adults and cognitively normal older adults for pictorial stimuli and perceptual priming tasks (Ballesteros et al., 2006, 2008). The present results suggest that selective attention at encoding has a significant influence not only in implicit perceptual memory tasks but also in this implicit conceptual memory task. The results also indicate that neither the young nor the older adults showed priming for unattended objects.

## EXPERIMENT 2: EXPLICIT MEMORY

A new set of 120 picture outlines was used in this explicit memory task. After completing the study phase in which they were asked to attend to the stimuli of a prespecified color and to remember them for a subsequent memory test, participants performed the old–new picture recognition task inside the scanner to evaluate the influence of attention at encoding on explicit memory. At test, the 72 pictures presented at study plus 48 new pictures were displayed one by one. The procedure used was similar to that of the implicit classification task. Only the test instructions changed.

### ENCODING PHASE

Participants were instructed to try to remember the stimuli presented in a given color (green or blue) while they performed the classification task. Half of the participants attended the blue picture outlines and the other half to the pictures with green outlines.

### TEST PHASE

At test, attended, unattended, and new stimuli were presented in black in a different random order for each participant. They indicated whether the picture was “old” or “new” by pressing one of two response buttons.

### RESULTS AND DISCUSSION

In the encoding phase, two young participants were eliminated due to technical problems and two older adults due to their low accuracy (below 75% correct). Twenty-two young adults and 18 cognitively normal older adults entered into the analyses. The mean number of errors for older adults during the encoding phase was 2.1 and for younger adults 1.4 (out of a maximum of 36). The univariate ANOVA conducted on errors as the dependent variable for group (young and older adults) showed that the main effect of group was not statistically significant [*F*(1,38) = 2.38, *p* > 0.05, MSE = 2.10, partial η^2^ = 0.05, 1-β error prob = 0.32]. The analysis showed that the two age groups were similarly accurate at encoding.

**FIGURE 5:**
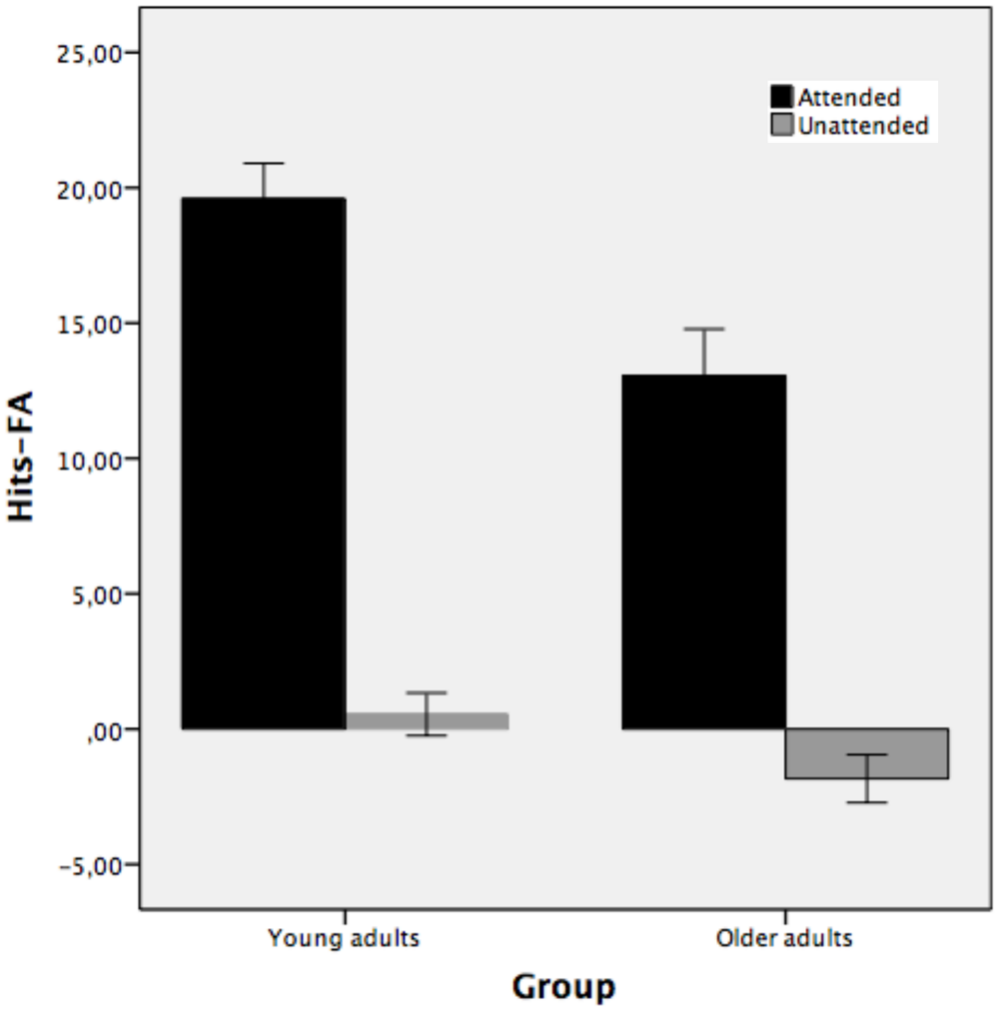
**Mean total number of hits-false alarms for recognized picture names as a function of study condition: studied attended, studied unattended pictures (Experiment 2).** Bars indicate ±1 SD from the mean.

**Figure 5** illustrates the recognition performance of young and older adults expressed in terms of the correct recognition memory of hits – false alarms using frequencies as a function of study condition. The results clearly show that there was recognition memory for those picture outlines that were attended at encoding but not for unattended stimuli.

The mixed-model ANOVA performed on the correct recognition measure of hits – false alarms as a function of study condition (attended and unattended) for young and older adults shows that the main effect of group was reliable [*F*(1,38) = 9.74, MSE = 40.36, *p* < 0.01, partial η^2^ = 0.20, 1-β error prob = 0.32]. Young adults recognized pictures as old or new better (10.06) than older adults (5.61). The main effect of study condition was also statistically significant [*F*(1,38) = 310.15, MSE = 18.37, *p* < 0.001, partial η^2^ = 0.89, 1-β error prob = 1]. Attended stimuli (*M* = 16.33) were recognized better than unattended stimuli (*M* = -0.64). The two-way group × study condition interaction was also reliable [*F*(1,38) = 4.65, MSE = 18.37, *p* < 0.05, partial η^2^ = 0.10, 1-β error prob = 0.98]. The interaction indicates that the difference between attended and unattended stimuli was greater for young adults (mean 19.59 and 0.54 for attended and unattended, respectively) than for older adults (mean 13.05 and -1.83 for attended and unattended, respectively). Both groups showed recognition for those objects that were attended at study but not for unattended objects. However, recognition for attended stimuli was higher in young than older adults, while the two groups did not differ in the unattended condition.

To investigate whether age differences in recognition reflect a difference in the hit rate, the false alarm rate or both, we conducted a mixed factorial ANOVA on hits as dependent variable, with group and study condition as factors. The analysis showed that the main effect of study was reliable [*F*(1,38) = 310.15, MSE = 18.37, *p* < 0.001, partial η^2^ = 0.89, 1-β error prob = 1]. A pairwise comparison showed that the mean of hits for the attended stimuli was 26.94 while for unattended stimuli it was 9.98. The main effect of group was not significant [*F*(1,38) = 0.53, MSE = 31.92, *p* > 0.05, partial η^2^ = 0.01, 1-β error prob = 0.05], suggesting that the two groups had a similar number of hits (18.93 and 18 for young and older participants, respectively). Finally, the two-way group × study condition interaction was significant [*F*(1,38) = 4.65, MSE = 18.37, *p* > 0.05, partial η^2^ = 0.16, 1-β error prob = 0.99]. This interaction showed that the two groups differed in the number of hits for attended stimuli (mean 28.45 and 25.44 for young and older adults, respectively) but not for the unattended stimuli (mean 9.40 and 10.55 for young and older adults, respectively). The ANOVA conducted with the number of false alarms as dependent variable showed that group was not significant [*F*(1,38) = 2.70, MSE = 45.49, *p* > 0.05, partial η^2^ = 0.06, 1-β error prob = 0.19]. The mean of false alarms was 8.86 and 12.38 for young and older participants, respectively.

The main finding of this experiment suggests that attention at encoding is critical for explicit memory and is in agreement with many previous findings showing that selective attention at encoding is required for explicit (episodic) memory (e.g., Rock and Gutman, 1981; Craik et al., 1996; Ballesteros et al., 2006, 2007).

## GENERAL DISCUSSION

The main results of the present study were as follows: (1) selective attention to a picture at encoding had profound effects on conceptual repetition priming, with significant conceptual priming for attended stimuli but not for unattended stimuli (Experiment 1); (2) selective attention to one or other picture outline had a great effect on explicit memory, as demonstrated by the good recognition of attended pictures and lack of recognition for the previously presented but unattended pictures (Experiment 2); (3) the two age groups showed the same level of conceptual facilitation for attended pictures and complete lack of facilitation for stimuli that had been presented at encoding but were not fully attended (Experiment 1); and (4) young adults had better explicit long-term recognition memory than older adults, but neither age group recognized the unattended objects (Experiment 2).

We found no evidence in the present study for negative priming (NP). In NP, the prime is a distractor and it is not the focus of attention. It is important to mention that NP occurs across successive trials, while repetition priming can last hours, days, and even months. NP is not observed when stimuli are presented alone at test, as in the present study. NP occurs when the time needed to name a selected object increases, as in the case of a previously unattended object (Tipper, 1985). The stimulus onset asynchrony (SOA) between prime and probe is very short in NP, while in our study it was much longer, which enabled us to record response times for each specific stimulus (Moore, 1994; Vuilleumier et al., 2005; Ballesteros et al., 2006, 2008).

### SELECTIVE ATTENTION AFFECTS BOTH IMPLICIT AND EXPLICIT MEMORY IRRESPECTIVE OF THE AGE OF PARTICIPANTS

Our findings are in contrast with early views that implicit memory is automatic and does not require attention at encoding (e.g., Eich, 1984; Parkin et al., 1990; Szymanski and MacLeod, 1996). They are in agreement with more recent findings that suggest the importance of attention in perceptual and conceptual repetition priming (e.g., Ballesteros et al., 2006, 2008, 2013a,b; Prull, 2013). Both types of priming require attention at encoding. A number of early studies that support the automaticity of implicit memory manipulated divided attention and not selective attention. These studies reported significant priming under divided attention conditions at encoding (e.g., Parkin and Russo, 1990; Parkin et al., 1990).

Older adults are more prone to distraction than young adults. It has been proposed that increased distractor processing reflects an age-related decline in a central inhibitory mechanism (Hasher and Zacks, 1988). Processing irrelevant information by older adults during encoding would reduce memory for targets, and the semantic content of the distraction would also contaminate memory (Ikier and Hasher, 2006; Weeks and Hasher, 2014 for a review). This might lead to the prediction that older adults would be less able to exclude distracting stimuli than young adults and that this would affect both the extent of priming and the pattern of recognition results. According to this hypothesis, less difference would be expected in priming and recognition for older than for young adults between attended and unattended pictures. This pattern of results does not fit with the present findings. There was no age difference in priming, as both young and older adults showed the same level of priming for attended objects and no priming for unattended ones. In recognition, the age difference appeared in the attended and not unattended items.

Both young and older participants in Experiment 1 were able to select by color and to attend selectively to one of two stimuli located close to the right and left of fixation. This finding is concordant with studies from our laboratory that used slightly different procedures and perceptual implicit memory tasks. For instance, experiments conducted with young adults (Ballesteros et al., 2006) using an overlapping picture procedure at encoding showed that the picture-fragment identification threshold was lower and object naming was faster for the attended than the unattended overlapping picture outlines (Experiments 1 and 2). Moreover, the study showed reliable implicit and explicit memory for attended but not for unattended pictures (Experiment 3). The present results are also in agreement with previous findings with young adults, cognitively normal older adults and AD patients (Ballesteros et al., 2008). In that study, reliable implicit memory for visually presented pictures and for haptically presented objects was found only for attended stimuli in both young and older adults. In contrast, AD patients did not show implicit memory for attended stimuli, suggesting an early deficit of selective attention in AD.

The present results are also in accordance with findings from a recent priming study (Ballesteros et al., 2013b) conducted with sedentary and physically active older adults. Both groups of older adults exhibited robust priming for attended pictures, which were identified at a lower threshold than unattended and new pictures. Interestingly, older adults with MCI did not show implicit memory for objects that were attended at encoding, whereas young and older cognitively normal adults did (Ballesteros et al., 2013c). As in the present study, unattended pictures at encoding did not produce facilitation, suggesting that repetition priming, as assessed either with a perceptual test (*picture fragment completion*) or with a conceptual task (*conceptual classification* as in the present study), is not automatic and requires attention at encoding. Presenting the pair of different colored stimuli at encoding either overlapping or side by side produced the same results; that is, attended repeated stimuli were named faster at test than new ones.

The complete “amnesia” for unattended picture outlines obtained in the present study in both the indirect implicit memory task and the recognition memory task is in agreement with the results of Vuilleumier et al. (2005) with young adults. They found that priming was reduced relative to attended pictures, but priming was significantly greater than for unattended pictures, whereas our young and older participants were “amnesic” for the unattended objects in the conceptual implicit memory task. However, methodological differences between Vuilleumier et al.’s (2005) study and the present one may explain the different results regarding the role of selective attention in implicit memory. Their young participants were shown pairs of overlapping pictures in one of two colors (one prespecified color was attended and the other was unattended) in rapid succession (250 ms duration with 250 ms blank intervals) at encoding. In our study, pairs of non-overlapping pictures were presented in each trial for a longer duration (1000 ms). The encoding tasks also differed, as their participants had to press a key in response to any infrequent non-sense shape, while ours performed a conceptual classification task and responded to each picture outline.

Our results are in agreement with those of a recent study that used verb generation, a conceptual implicit memory task (Prull, 2013). That study showed that conceptual priming is sensitive to attention at encoding and is also in disagreement with the automatic encoding view of implicit memory, because several experiments demonstrated that divided attention reduces verb generation priming while selective attention extinguished it totally.

Using a category verification task, Mulligan and Peterson (2008) found that implicit memory was not sensitive to selective attention manipulations (Experiment 7a). Although their task differed from the semantic classification task employed in the present study, both tasks require conceptual analysis of stimuli at test. Based on their results, they concluded that category verification is unaffected by a selective attention manipulation that impairs other implicit tests. Several differences between Mulligan and Peterson’s (2008) study and ours could explain the results, including differences in the task used (category verification *vs*. semantic classification) and the stimulus type (words *vs.* pictures). Another possible difference appears to be the presence vs. absence of possible stimulus-response (S-R) learning effects. The S-R learning effects have been related with the formation and retrieval of direct bindings between the stimulus and the prior responses elicited by that stimulus. This effect has been proposed as an alternative origin of priming (Horner and Henson, 2009). Moreover, Mulligan and Peterson (2008) presented individual words in different colors and participants either had to identify the color or name the word. In the present study, participants were presented with two picture outlines and attended to one of the pictures. When presented with a word, the participant forms a mental image of a specific exemplar of the object. Phonological representations are also automatically accessed when presented with words (Bowers and Turner, 2002). In contrast, a picture provides direct access to an exemplar. It is possible that the double access of words (mental image and phonological representations) contributed to the lack of sensitivity to selective attention manipulations.

### YOUNG ADULTS HAVE BETTER EXPLICIT MEMORY THAN OLDER ADULTS BUT SIMILAR PERCEPTUAL AND CONCEPTUAL IMPLICIT MEMORY FOR ATTENDED PICTURES

The present findings of spared repetition priming effects for attended objects coupled with reduced explicit recognition is consistent with age-related differences in priming and explicit memory reported in many previous studies in which just one stimulus (always attended) was presented at encoding (e.g., Mitchell and Bruss, 2003; Lustig and Buckner, 2004; Soldan et al., 2008; Bergerbest et al., 2009; Osorio et al., 2009, 2010; Sebastián et al., 2011; Sebastián and Ballesteros, 2012; Ballesteros et al., 2013a). Interestingly, this dissociation between these memory functions in young adults (Cooper et al., 1992; Reales and Ballesteros, 1999), older adults (Soldan et al., 2008; Bergerbest et al., 2009) and AD patients (Ballesteros and Reales, 2004; Ballesteros et al., 2007) supports the idea that implicit and explicit memory tasks are differentially sensitive to aging (see, Tulving and Schacter, 1990; Squire, 2004).

The lack of an age difference in priming could be due to the fact that the same natural/artificial judgment task was performed at both encoding and test. In conjunction with conceptual facilitation due to stimulus repetition, a potential motor process or S-R mapping could have led to the observed priming (Horner and Henson, 2009; see Henson et al., 2014, for a recent review). In fact, Dew and Giovanello (2010) found that the ability to encode an association between a stimulus and its task-specific response is preserved in older adults. However, in a previous perceptual repetition priming study (Ballesteros et al., 2008) young and older participants performed an *object-naming* task at encoding and a *picture fragment completion* task at test, with attended, unattended and new stimuli. The results showed similar priming in young and older adults for attended but not for unattended pictures. It would be interesting in future studies to investigate the effects of attention at encoding in young and older participants when performing a different task at study and at test, and when the response to a repeated stimulus switches from a manual (e.g., key press) to a vocal (e.g., yes/no) response, or vice versa. Stimulus-decision or stimulus-task associations can be learned too, all of which may play a role in this study (Horner and Henson, 2009).

## CONCLUSION

In sum, the results of the present study suggest that selective attention at encoding influenced behavior and has profound effects for visual stimuli presented closely to the left or right of fixation at encoding. Using the same type of stimuli and same experimental procedure at encoding and test and changing only the test instructions for the implicit and explicit memory tasks, the present study shows that both types of long-lasting memory require attention at encoding, but that while conceptual implicit memory for attended pictures is similar in young and older adults, younger adults have superior explicit recognition for these pictures than cognitively normal older adults.

## Conflict of Interest Statement

The authors declare that the research was conducted in the absence of any commercial or financial relationships that could be construed as a potential conflict of interest.
